# Insights into pegRNA design from editing of the cardiomyopathy‐associated phospholamban R14del mutation

**DOI:** 10.1002/1873-3468.70097

**Published:** 2025-06-24

**Authors:** Bing Yao, Qiangbing Yang, Christian J.B. Snijders Blok, Mark A. Daniels, Pieter A. Doevendans, Raymond Schiffelers, Joost P.G. Sluijter, Zhiyong Lei

**Affiliations:** ^1^ Experimental Cardiology Laboratory, Department of Cardiology, Division of Heart and Lungs University Medical Center Utrecht the Netherlands; ^2^ Regenerative Medicine Center Utrecht, Circulatory Health Research Center University Medical Center Utrecht, University Utrecht the Netherlands; ^3^ CDL Research University Medical Center Utrecht the Netherlands; ^4^ Netherlands Heart Institute (NLHI) Utrecht the Netherlands; ^5^ Central Military Hospital (CMH) Utrecht the Netherlands

**Keywords:** gene therapy, pegRNA optimization, *Phospholamban*, *PLN* R14del mutation, prime editing

## Abstract

Prime editing (PE) represents a transformative genome‐editing technology and enables precise insertions, deletions, and base substitutions without introducing double‐strand breaks, thereby reducing undesired indels and off‐target effects. Despite advancements in enhanced prime editors and optimized prime editing guide RNAs (pegRNAs), designing effective pegRNAs remains a major challenge. The phospholamban (*PLN*) R14del mutation is associated with cardiomyopathies, making it a crucial target for precise gene‐editing strategies. In this study, we explored pegRNA features that contribute to high editing efficiency using the FluoPEER.*PLN* R14del reporter cell line. Through systematic screening, we identified three pegRNAs with significantly enhanced editing efficiency. Our findings underscore the importance of pegRNA secondary structure and stability in optimizing prime editing, providing valuable insights into precise gene correction strategies.

## Graphical abstract

This study reveals how prime editing guide RNA (pegRNA) secondary structure and reverse transcriptase template length affect prime editing efficiency in correcting the phospholamban R14del cardiomyopathy‐associated mutation. Insights support the design of structurally optimized enhanced pegRNAs for precise gene therapy.

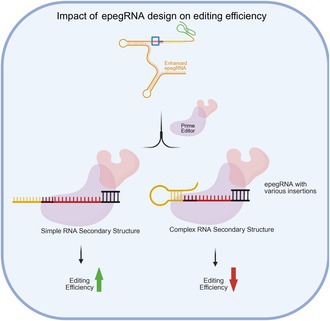

## Abbreviations

Cas9 CRISPR‐associated protein 9

DCM dilated cardiomyopathy

ddPCR droplet digital polymerase chain reaction

DSB double‐strand break

epegRNA enhanced prime editing guide RNA

FACS fluorescence‐activated cell sorting

FBS fetal bovine serum

HEK293FT human embryonic kidney 293FT cells

iPSC‐CMs induced pluripotent stem cell‐derived cardiomyocytes

M‐MLV RT moloney murine leukemia virus reverse transcriptase

MMR mismatch repair

mpknot engineered RNA structural motif (MP‐knot)

PAM protospacer adjacent motif

PBS primer binding site

PCR polymerase chain reaction

PE prime editing

pegRNA prime editing guide RNA

*PLN* phospholamban

R14del arginine 14 deletion (a mutation in PLN)

RTT reverse transcriptase template

sgRNA single‐guide RNA

tevopreq engineered RNA structural motif (TevopreQ1)

UNAFold UNAfold RNA secondary structure prediction software

WT wild type

Prime editing (PE) has emerged as a next‐generation genome‐editing platform that addresses limitations inherent to CRISPR‐based tools, including clustered regularly interspaced short palindromic repeats (CRISPR), adenine base editors (ABEs), and cytosine base editors (CBEs) [1]. While CRISPR‐Cas9 enables targeted DNA cleavage, it often leads to unintended indels due to reliance on double‐strand breaks (DSBs) and donor templates [2]. Base editors (BEs) facilitate single‐nucleotide modifications but are constrained by their inability to introduce multi‐nucleotide edits [3, 4]. By contrast, PE enables precise insertions, deletions, and base substitutions while avoiding DSBs, significantly reducing off‐target effects and rendering it a promising therapeutic tool [5].

PE has undergone iterative optimization to enhance its efficiency and stability. The original PE1 system comprises a fusion protein combining *Streptococcus pyogenes* Cas9 H840A nickase with wild‐type Moloney murine leukemia virus (M‐MLV) reverse transcriptase (RT) and a prime editing guide RNA (pegRNA) [5]. The pegRNA consists of a primer binding site (PBS), a reverse transcriptase template (RTT), and the desired edit sequence. Upon recognizing the protospacer adjacent motif (PAM) at the target site, the PE complex nicks the DNA, facilitating hybridization of the PBS and enabling RT to synthesize the edited DNA sequence, which is subsequently integrated into the genome [5]. Further modifications of PE have enhanced its efficiency; while PE2 replaces the wild‐type RT with an engineered variant, PE3 introduces a secondary single‐guide RNA (sgRNA) to nick the non‐edited DNA strand [5]. PE4 and PE5 incorporate mismatch repair (MMR) inhibition via a dominant‐negative MLH1 protein, significantly improving editing efficiency [6]. Additionally, PE6 enhances activity through protein evolution and codon optimization [7], while PE7 utilizes the RNA‐binding La protein to stabilize pegRNAs, further boosting efficiency [8]. Despite these advancements, pegRNA design remains a significant bottleneck in prime editing efficiency. Factors influencing pegRNA efficacy include PBS and RTT lengths, secondary structure stability, and susceptibility to degradation [9, 10, 11]. Modifications such as G‐quadruplex (G‐PE) [12], xrRNA‐modified pegRNAs [13], and enhanced pegRNAs (epegRNAs) incorporating RNA structural motifs (evopreQ1 and mpknot) have demonstrated improved editing efficiency [14].

The phospholamban (*PLN*) R14del mutation is a pathogenic variant linked to inherited cardiomyopathy, which contributes to the development of dilated cardiomyopathy (DCM) and heart failure [9, 10]. This mutation arises from the deletion of arginine at position 14 in phospholamban, leading to disrupted calcium homeostasis in cardiomyocytes [11, 15]. Given its clinical significance, the development of precise and efficient gene‐editing strategies to correct *PLN* R14del is essential for potential therapeutic applications.

To systematically investigate pegRNA design parameters, we established a reporter cell line incorporating the *PLN* R14del mutation. This model enabled direct visualization and quantification of the editing efficiency of the *PLN* R14del mutation via mCherry fluorescence. Through pegRNA screening, we identified three highly efficient candidates to correct the *PLN* R14del mutation and analyzed their sequence characteristics and secondary structure features using predictive modeling. Our results elucidate the role of pegRNA structural stability in optimizing prime editing and inform strategies for precise gene corrections.

## Method and materials

### Plasmid cloning

FluoPEER.*PLN* R14del reporter plasmid was cloned using the previous reported backbone of the pLV(64)‐CMV‐eGFP‐P2A‐KO plasmid [16]. This plasmid was cleaved directly in the middle of the eGFP and mCherry sequences with SrfI (#R0629S; New England Biolabs, Ipswich, MA, USA), for 2 h at 37 °C, creating a blunt end, after which the 10 kb fragment was isolated from the gel. The cleaved backbone and PCR amplicons containing the *PLN* R14del mutation sequence and two overhangs at both the 5′‐ end and 3′‐ end were annealed and inserted using the NEBuilder® HiFi DNA Assembly protocol (#E2621; New England Biolabs, USA). All primers used are listed in Table S1 and were synthesized by Integrated DNA Technologies (IDT, Coralville, IA, USA).

Cloning of epegRNA plasmids was performed according to the previously described protocols [5, 14]. In brief, oligonucleotide duplexes of the epegRNA spacer, epegRNA extension, and epegRNA scaffold sequences were ordered, containing the appropriate overhangs, and subsequently annealed. The annealed epegRNA duplexes were ligated into the pU6‐tevopreq1‐GG‐acceptor using Golden Gate assembly with BsaI‐HFv2 (#R3733S; New England Biolabs, USA) and T4 DNA ligase (#M0202S; New England Biolabs, USA) following a protocol of 30 cycles of 5 min at 16 °C and 5 min at 37 °C. All sequences used are listed in Table S2 and were synthesized by IDT.

pU6‐tevopreq1‐GG‐acceptor (Addgene, #174038), pCMV‐PE2 (Addgene, #132775), pCMV‐PEmax (Addgene, #174820), pCMV‐PE2RnaseH^del^ (pCMV‐PEmaxRNaseHtrunc, Addgene, #207858), and pCMV‐PE4 (pCMV‐PEmax‐P2A‐hMLH1dn, Addgene, #174828) were obtained from Addgene.

### Polymerase chain reaction (PCR)

For the construction of the FluoPEER.*PLN* R14del reporter plasmid, a PCR was performed using *PLN* R14del mutation homozygous genomic DNA as the template. The reaction mixture included 50 ng of genomic DNA, 1 μL of each primer (10 mm), 12.5 μL of Q5 Hot Start High‐Fidelity 2× Master Mix (#M0494; New England Biolabs, USA), resulting in a final reaction volume of 25 μL by RNase‐free water. The PCR was performed in a C1000 Touch thermal cycler (BioRad Laboratories, Hercules, CA, USA).

For epegRNA PCR amplicon, 1 μL of plasmid, 1 μL of each primer (10 mm), 10.5 μL of Rnase‐free water, and 12.5 μL of Q5 Hot Start High‐Fidelity 2× Master Mix (M0494; New England Biolabs, USA) were used in a final reaction volume of 25 μL performed in a C1000 Touch thermal cycler (BioRad Laboratories, USA). PCR products were run on a 1% agarose gel containing ethidium bromide solution at a 1 : 10 000 dilution (#21–1 056 334; Invitrogen, Carlsbad, CA, USA) and imaged in the ChemiDoc MP imaging system (BioRad Laboratories, USA). Sanger sequencing was performed by Macrogen. We designed epegRNA +AG, +AGA, and + CGC constructs. The +AG insertion was introduced to induce a frameshift mutation, enabling the detection of highly efficient pegRNAs via the FluoPEER.*PLN* R14del reporter cell system. In contrast, the +AGA insertion represents the actual correction of the PLN R14del mutation, restoring the correct reading frame. The +CGC insertion was designed as a substitution for AGA while preserving the same amino acid sequence. This design allows us to evaluate both screening efficiency and the precise correction of the target mutation. All primers are shown in Table S1 and were synthesized by IDT.

### Cell culture, lentiviral production, and lentiviral transduction

HEK293FT cells (RRID: CVCL_6911) were obtained from Thermo Fisher Scientific and cultured in Dulbecco's modified Eagle medium (DMEM, 10564011; Thermo Fisher Scientific, Waltham, MA, USA) with 100 μg·mL^−1^ streptomycin, 100 U·mL^−1^ penicillin (15 140 122; Thermo Fisher Scientific, USA), 10% fetal bovine serum (FBS, MFCD00132239; Sigma‐Aldrich, St. Louis, MO, USA) at 37 °C, and 5% CO_2_.

HEK293FT was authenticated by short tandem repeat (STR) profiling within the past three years. All experiments were conducted using cells that were confirmed to be free of mycoplasma contamination using the MycoAlert™ Mycoplasma Detection Kit (Lonza, Walkersville, MD, USA).

For lentivirus production, HEK293FT cells were plated in a T175 flask at a 50–60% density. After 24 h, the cells were transfected with a mixture containing 20 μg of the pLV(64)‐CMV‐eGFP‐P2A‐*PLN* R14del plasmid, 10 μg of pCMV‐VSVG, 10 μg of pCMV‐deltaR8.74 (Addgene, #22036), and 120 μL of polyethylenimine (1 mg·mL^−1^). The medium from virus‐producing cells was collected and filtered through a 0.45 μm filter to remove cell debris and large contaminants. To each 10 mL of the filtered medium, 2.5 mL of 50% PEG8000, 1 mL of 4 m NaCl, and 1.1 mL of PBS were added. The mixture was then incubated at 4 °C overnight for virus precipitation. After incubation, the mixture was centrifuged at 7000–7500 ***g*** for 30 min at 4 °C, and the resulting white pellet containing the precipitated virus was carefully collected. The pellet was resuspended in 100 μL of 50 mm Tris/HCl buffer (pH 7.4) and aliquoted into 10 μL per tube. These aliquots were stored at −80 °C for future use.

The polybrene stock solution (TR‐1003‐50UL; Thermo Fisher Scientific, USA) was diluted using the culture medium to achieve a final concentration of 8 ng·μL^−1^. This diluted polybrene was added to the HEK293FT cells along with the lentivirus containing the transgene. On the second day post‐infection, the culture medium was changed to remove polybrene and virus. The cells were then cultured until the fourth day to ensure successful transduction, and single cells were sorted at 14 days by Multi‐Application Cell Sorter (MA900; Sony Biotechnology, San Jose, CA, USA). To prepare for sorting, the cells are resuspended in PBS. Once the MA900 Multi‐Application Cell Sorter is powered on and calibrated according to the manufacturer's instructions, a suitable nozzle is selected to minimize shear stress. Instrument settings for forward scatter, side scatter, and the FITC fluorescence channel is optimized. Following calibration, the gating strategy excludes debris, doublets, and dead cells, thus isolating the population of interest (e.g., FITC^+^‐positive events). The sorter is then configured to deposit single cells into individual wells of a 96‐well plate. Immediately after sorting, an appropriate volume (100–200 μL) of pre‐warmed complete medium is added to each well, and the plate is returned to a 37 °C, 5% CO_2_ incubator. The single‐cell clones are monitored over one to two weeks, with medium changes or top‐ups every two to three days as necessary. When individual clones have expanded sufficiently, they are transferred to larger vessels for further analysis, such as genotyping.

### Transfection of FluoPEER. *PLN* R14del reporter cells

Transfection experiments were performed in 12‐well plates, with transfections carried out at a density of 300 000 cells/well after 24 h of seeding. For transfections, 0.75 μg of each prime editor plasmid and 0.25 μg of epegRNA PCR amplicons were used, following the manufacturer's protocol of Lipofectamine 3000 Transfection Reagent (#L3000001; Invitrogen, USA). After 72 h, cells were detached and centrifuged for 5 min at 300 × g, then resuspended in 200 μL PBS. Cells were imaged by Invitrogen EVOS FL Digital Inverted Fluorescence Microscope (Thermo Fisher Scientific, USA), performed by LSRFortessa™ X‐20 (BD Biosciences, San Jose, CA, USA).

### Droplet digital PCR

Genomic DNA was extracted from FluoPEER.PLN R14del reporter cells transfected with epegRNAs using the DNeasy Blood and Tissue Kit (#69504; Qiagen, Germany). For droplet digital PCR (ddPCR), the primers and probe mixture for the RPP30 assay (#12003805) and PLN R14del assay (#12002312; BioRad Laboratories, USA) were obtained from BioRad. The reaction mixture included 50 ng of genomic DNA, 1 μL of 20X Probe assay, and 11 μL of ddPCR Supermix for Probes (No dUTP) (186–3023; BioRad Laboratories, USA), supplemented with nuclease‐free water to a final volume of 22 μL. Droplets were generated using the QX200 Automated Droplet Generator (BioRad, Germany) according to the manufacturer's instructions. PCR amplifications were performed in a thermal cycler (BioRad, Germany) under the following conditions: 98 °C for 1 min; 40 cycles of 98 °C for 10 s, 57 °C for 30 s, and 72 °C for 60 s, followed by 72 °C for 5 min. Samples were measured and analyzed using the QX200 Droplet Reader (BioRad, Germany).

### Data analysis

Sanger sequencing chromatograms were made in Snapgene Viewer. Flow cytometry data were analyzed using flowjo Software. Stability and structural analysis of epegRNA was predicted by UNAFold (http://www.unafold.org/mfold/applications/rna‐folding‐form.php). All figures were made in Prism (graphpad Software). Data are presented as the mean ± standard deviation (SD) unless otherwise specified. For comparisons between two groups, a two‐tailed Student's *t*‐test was used. For multiple group comparisons, one‐way or two‐way analysis of variance (ANOVA) was performed. Statistical significance was defined as *P* < 0.05, and levels of significance are denoted as follows: *P < 0.05, **P < 0.01, ***P < 0.001. All experiments were conducted with at least three biological replicates, and each assay was performed in triplicate to ensure reproducibility and reliability.

## Results

### Generation of FluoPEER. *PLN* R14del reporter cell for pegRNA screening

**Fig. 1:**
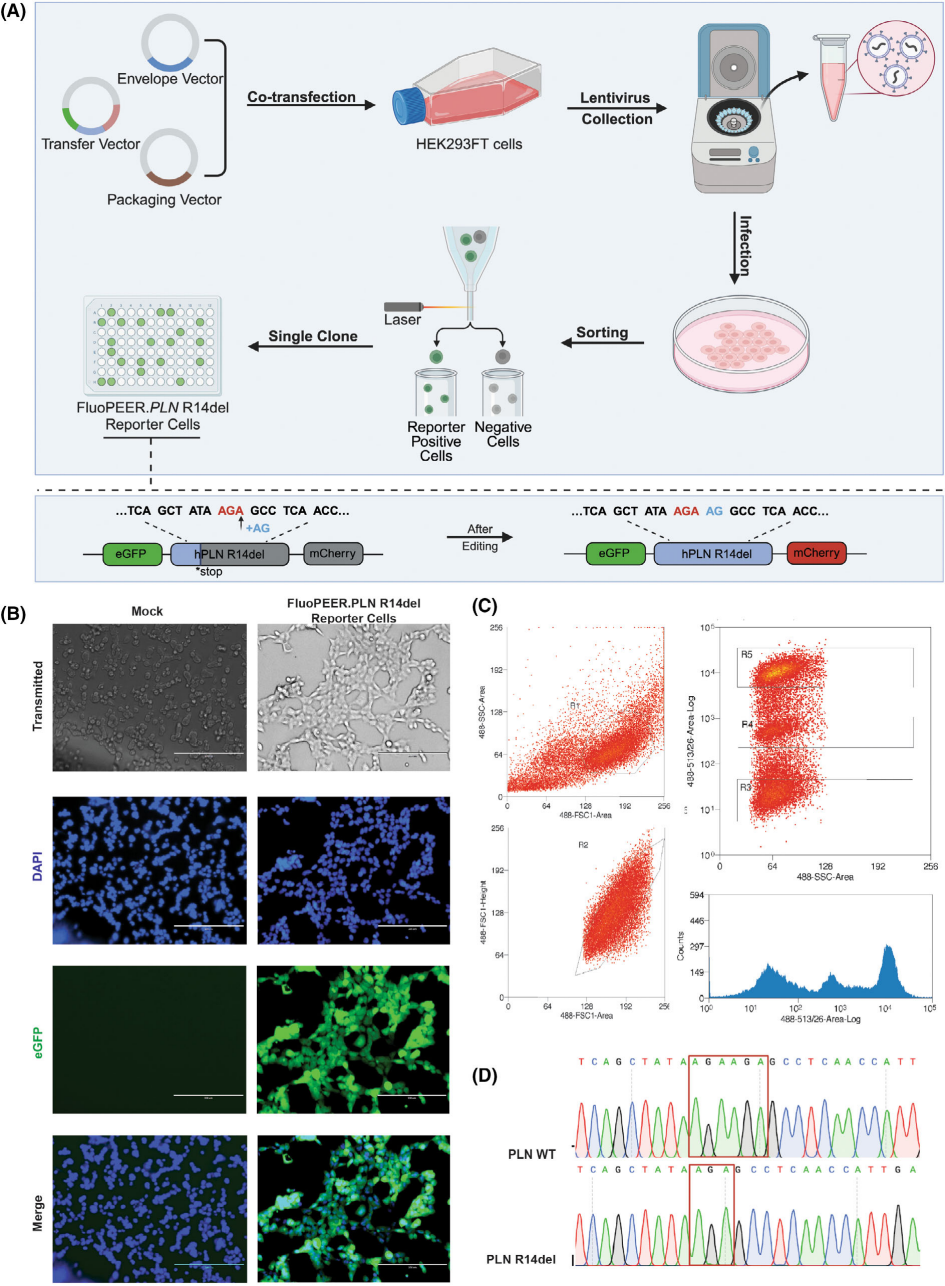
Schematic representation of the construction of FluoPEER.*PLN* R14del reporter cells. (A) The process includes producing lentivirus containing interest gene sequence and infecting HEK293FT cells, which are subsequently sorted to isolate single clones. GFP‐positive and GFP‐negative cells are identified using fluorescence‐activated cell sorting (FACS). Design of the FluoPEER.*PLN* R14del reporter construct: The reporter system includes an eGFP gene upstream and an mCherry gene downstream of the *PLN* R14del sequence. A 2 bp insertion (+AG) at the *PLN* R14del site is shown, which allows the detection of editing events via frameshift correction in the reporter cells as mCherry‐positive signal. (B) Validation of FluoPEER.*PLN* R14del reporter cells. Transmitted light and fluorescence microscopy images show the expression of eGFP and DAPI‐stained nuclei in both FluoPEER.*PLN* R14del reporter cells. Scale bars represent 200 μm. (C) Flow cytometry analysis of FluoPEER.*PLN* R14del reporter cells. Scatter plots illustrate the distribution of cells based on fluorescence intensity, distinguishing eGFP‐positive (transfected) and eGFP‐negative (non‐transfected) populations. R1, total cell population. R2, single cells excluding debris and doublets. R3, cells with low eGFP expression. R4, cells with moderate eGFP expression. R5, cells with high eGFP expression. (D) Sequencing results of the *PLN* wild‐type (WT) and FluoPEER.*PLN* R14del reporter cell sequences. The red boxes indicate the location of the R14 deletion mutation within the *PLN* gene sequence.

To assess the editing efficiency of the designed epegRNAs, we established FluoPEER.*PLN* R14del reporter cells in HEK293FT cells via lentiviral transduction (Fig. 1A) [16]. Evaluating editing efficiency is essential to verify that the designed epegRNAs can precisely induce the intended genetic modifications. To generate these reporter cells, we first engineered a lentiviral vector carrying the FluoPEER.*PLN* R14del sequence, which was subsequently transduced into HEK293FT cells. In these reporter cells, eGFP is constitutively expressed (Fig. 1B), serving as a stable baseline marker for successful transduction. mCherry expression will be conditionally activated only upon a 2‐bp insertion, which leads to a frameshift mutation. Initially, we aimed to introduce a 3‐bp insertion to maintain the reading frame; however, a 2‐bp insertion was ultimately chosen as it effectively disrupts the open reading frame, ensuring a functional frameshift mutation and robust fluorescence‐based readout of successful editing events.

Following lentiviral transduction, single‐cell sorting was performed to isolate individual positive clones for the reporter. A cell clone exhibiting moderate eGFP expression levels was specifically selected (Fig. 1C, R4) to ensure experimental consistency by avoiding excessively high or low eGFP expression, which could introduce variability. The presence of the *PLN* R14del mutation in the sorted cells was subsequently validated through Sanger sequencing (Fig. 1D), confirming that the selected cells harbored the desired genetic modification. These validated cell lines were then expanded and utilized for downstream pegRNA screening and optimization experiments.

### Design and screening for epegRNA for *PLN* R14del correction using prime editing

PegRNA, a key component of the prime editing system, plays a crucial role in determining editing efficiency [5]. Various factors, including the lengths of the PBS and RTT sequences, significantly impact pegRNA performance [5, 17, 18]. Beyond these parameters, the structural integrity of pegRNA is also critical, encompassing elements such as the scaffold interacting with Cas9 and the overall stability of the pegRNA [19].

Previous reports indicate that editing efficiency can be dramatically enhanced by modifying the fourth thymine (T) in a continuous T sequence to adenine (A). This continuous T sequence may function as a pause signal for RNA polymerase III [20], potentially reducing transcription efficiency of the sgRNA. By mutating the T at position 4, this pause signal is disrupted, leading to increased sgRNA transcription efficiency. This mutation is also suggested to influence sgRNA structure, thereby enhancing its binding affinity to the Cas9 protein and improving its activity. Additionally, incorporating an extra RNA motif called “tevopreq”, which forms a loop structure to slow down pegRNA degradation, has been demonstrated to increase editing efficiency by 3–4 fold compared to standard pegRNA [14].

**Fig. 2:**
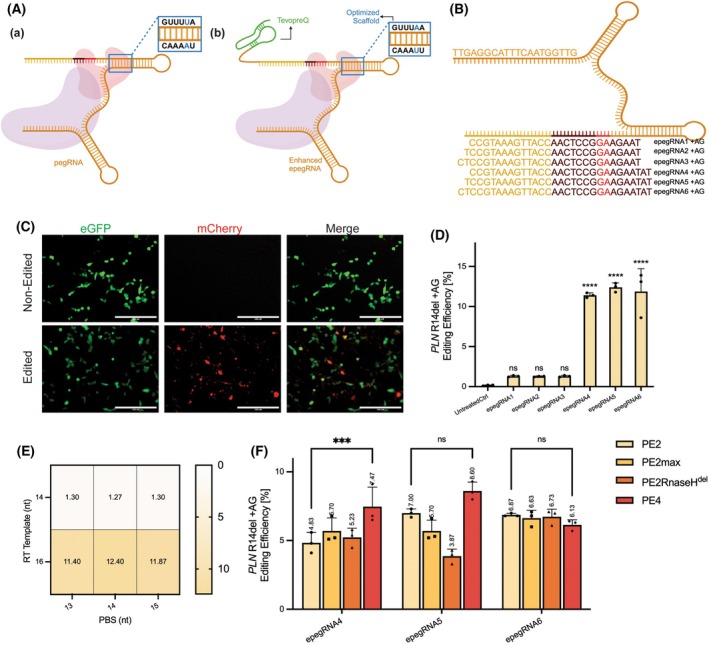
Evaluation of the enhanced epegRNA and Prime Editor Variants for the +AG insertion of *PLN* R14del Mutation. (A) Schematic illustrations of different pegRNA and epegRNA designs. (a) Standard pegRNA structure with Cas9 nuclease and reverse transcriptase domains. (b) Enhanced epegRNA with a base pair difference (highlighted in blue) in scaffold sequence and incorporating a loop structure (TevopreQ, highlighted in green) for improved stability and efficiency. (B) Schematic of epegRNAs designed for the *PLN* R14del target, showing the specific sequences in the PBS (yellow) and RT template (brown) regions. The inserted bases are highlighted in red. (C) Fluorescence microscopy images showing eGFP and mCherry expression in non‐edited and edited cells (transfected with epegRNA4 + AG). eGFP fluorescence (green) represents the intrinsic color of the reporter cells, while mCherry fluorescence (red) is activated upon successful editing. Scale bars represent 200 μm. (D) Editing efficiency for the *PLN* R14del + AG target using different pegRNAs (epegRNA1‐6) with the PE2 system. (***P < 0.0001, ns: not significant). Data are represented as mean ± SD (*n* = 3). (E) Heatmap representing editing efficiency based on varying primer binding site (PBS) lengths (13–15 nucleotides) and reverse transcriptase template (RT Template) lengths (14, 16 nucleotides). (F) Comparative analysis of editing efficiencies using different PE variants with epegRNA4, epegRNA5, and epegRNA6. The graph highlights significant differences in efficiency across the editor variants, with statistical significance indicated (***P < 0.001, ns: not significant). Data are represented as mean ± SD (*n* = 3).

In our study, we combined these two optimizations by introducing the tevopreq motif at the 3′ end of the mutant scaffold pegRNA to enhance its editing capability (Fig. 2A). We employed PCR amplification to generate epegRNAs, including only the U6 promoter and the epegRNA sequence (Fig. 2B, and Fig. S1A). This approach reduces extraneous vector sequences that may lead to unintended editing, thereby improving editing specificity.

Following this, we co‐transfected PE2 with each epegRNA PCR product into FluoPEER.*PLN* R14del reporter cells. Our results demonstrated that, when PBS length was held constant, epegRNAs with RTT lengths of 16 nt exhibited significantly higher editing efficiency (Fig. 2C,D), on average, 9.22‐fold greater than those with RTT lengths of 14 nt. Specific increases of 8.77‐, 9.76‐, and 9.13‐fold were observed across the three tested epegRNAs (Fig. 2E). These statistically significant findings highlight a clear advantage of using an extended RTT sequence to enhance prime editing efficiency. Based on these results, we selected three epegRNAs with 16‐nt RTTs (epegRNA4, 5, and 6) for further optimization.

### Evaluation of PE variants for effective +AG for editing *PLN* R14del

With the increasing demand for high editing efficiency in prime editing, multiple PE variants have been developed to enhance performance [21]. These variants aim to improve various aspects of the prime editing system, making it more efficient and adaptable for diverse applications. In this study, we assessed the capabilities of four prime editing variants, including PE2, PE2max, PE2RnaseH^del^, and PE4, in facilitating the +AG insertion for *PLN* R14del mutation modification.

PE2max exhibits increased nicking activity, which may enhance prime editing efficiency by promoting DNA nicking [6]. PE2RnaseH^del^, in contrast, features a reduced protein size, which is advantageous for vector‐based delivery, particularly in systems constrained by packaging limits, such as viral vectors [22, 23]. PE4 enhances editing efficiency by suppressing the cellular mismatch repair (MMR) pathway, which can otherwise correct the intended edits and reduce overall editing efficacy [6].

To compare the efficiency of these variants, we repeated the transfection process in FluoPEER.*PLN* R14del reporter cells. Our results revealed that all tested variants exhibited comparable editing efficiencies for the +AG insertion in *PLN* R14del mutation (Fig. 2F). Notably, only epegRNA4 showed a difference between PE4 and PE2, suggesting a potential advantage of PE4 in this specific context. However, since this trend was not consistently observed in other groups, the difference was not considered statistically significant across PE variants in *PLN* R14del correction.

These findings suggest that while PE4 may offer selective benefits under certain conditions, the choice of PE variant should be guided by the specific requirements of the PLN R14del editing application rather than a presumed overall superiority. Based on these results, we ultimately selected the combination of PE4 and epegRNAs for *PLN* R14del mutation correction (+AGA insertion), leveraging PE4's ability to suppress the MMR pathway, which could enhance the stability of the introduced edits.

### Comparison of +AG vs. +AGA insertions for editing *PLN* R14del

The correction of the *PLN* R14del mutation requires the insertion of the nucleotide sequence AGA following the codon for the 13th arginine residue (R). During initial epegRNA screening, we opted for an +AG insertion instead of +AGA in FluoPEER.*PLN* R14del reporter cells, as the +AG insertion induces a frameshift mutation. We hypothesized that this variation would not significantly affect editing efficiency, allowing for a more straightforward screening process. While the FluoPEER.*PLN* R14del reporter cells rely on the frameshift mutation caused by the +AG insertion, the +AGA insertion does not induce a frameshift and therefore could not be directly assessed in the same system. To circumvent this limitation, we employed ddPCR to quantify editing efficiency by comparing the ratio of the *PLN* R14del allele to the reference gene RPP30.

The results from ddPCR and FACS analyses indicated no significant differences in editing efficiency across epegRNA4‐6 (+AG insertions), validating that ddPCR serves as a reliable alternative to FACS for evaluating editing efficiency (Fig. S1B,C). After identifying epegRNA4, epegRNA5, and epegRNA6 as high‐efficiency candidates for +AG insertion, we redesigned these epegRNAs by substituting the original +AG insertion with the +AGA insertion necessary for *PLN* R14del mutation correction (Fig. S1D,E).

**Fig. 3:**
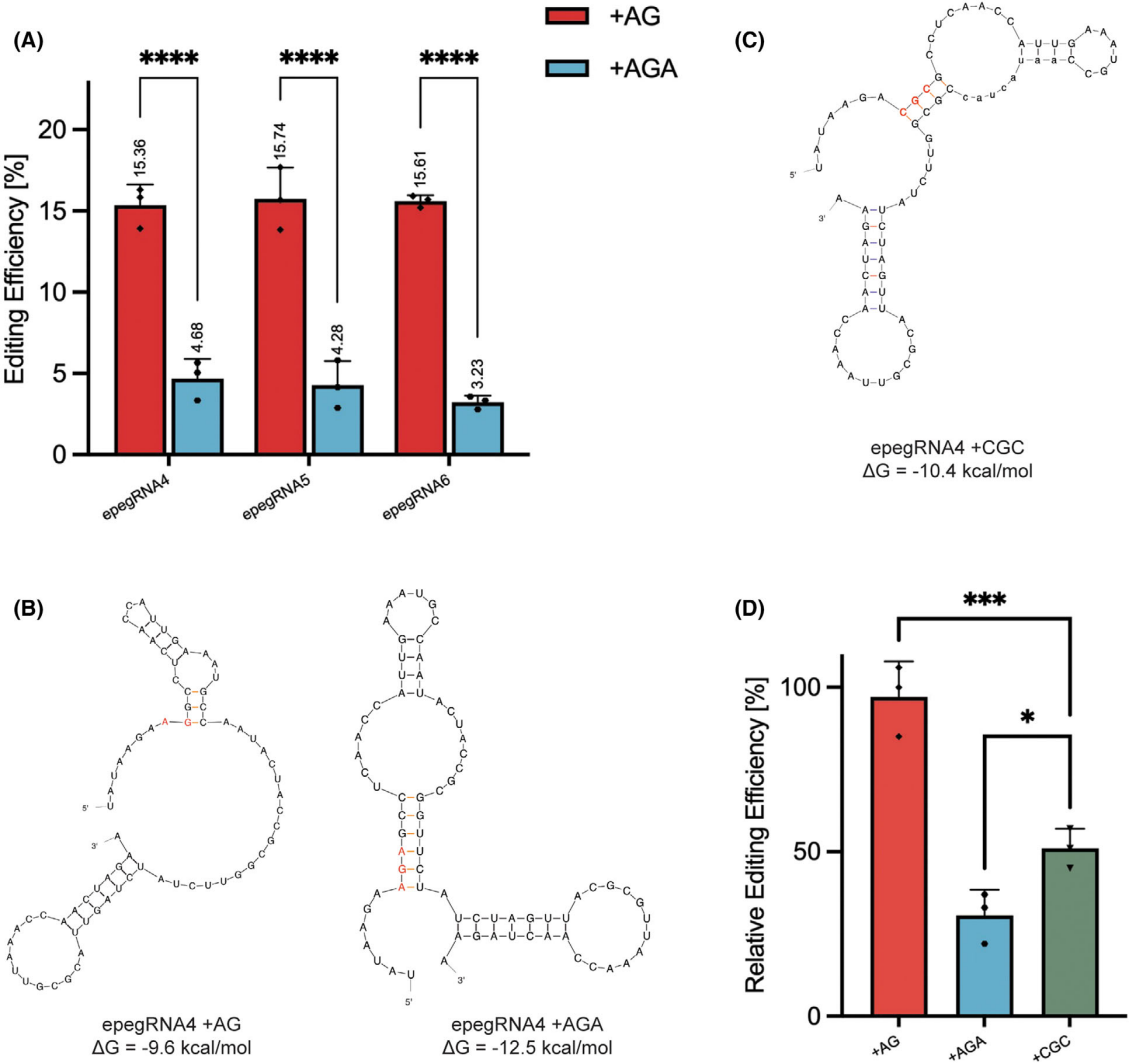
Impact of RNA Secondary Structure on Editing Efficiency of epegRNAs with the 2 bp and 3 bp Insertions. (A) Editing efficiencies (%) of epegRNA4, epegRNA5, and epegRNA6 in the presence of +AG (red) and + AGA (blue) insertions, measured as the percentage of successfully edited targets. Each bar represents the mean ± SD with individual data points shown. Statistical significance is indicated (****P < 0.0001). Data are represented as mean ± SD (*n* = 3). (B and C) The UNAFold‐predicted secondary structure and free energy (Δ*G*) of 3'extensions are shown. The inserted nucleotides are indicated in red, and the orange regions represent stem structures that are predicted to form near to the desired edits. (D) Relative editing efficiency of epegRNA4 with +AG, +AGA, and + CGC insertions, normalized to the +AG (set at 100%). Statistical significance is denoted (*P < 0.05) between +AGA and + CGC insertions. Data are represented as mean ± SD (*n* = 3).

Following co‐transfection with PE4, we observed correction efficiencies of 4.68%, 4.28%, and 3.23% for epegRNAs incorporating the +AGA insertion. This represents a marked reduction in editing efficiency compared to their +AG counterparts (Fig. 3A). Specifically, the efficiency differences were 10.68%, 11.46%, and 12.38%, respectively. The substantial decline in editing efficiency prompted further investigation to elucidate the underlying factors contributing to this reduction.

### RNA structural analysis identifies more stable structures in low‐efficiency epegRNAs

It has been reported that in the CRISPR‐Cas9 system, the free energy (Δ*G*) of the gRNA plays a crucial role in unzipping the DNA duplex, forming the RNA/DNA hybrid, and promoting Cas9 cleavage activity [24, 25]. Similarly, in the prime editor system, the free energy of the pegRNA may influence editing efficiency through these same mechanisms. To ensure the accuracy of Δ*G* values for each epegRNA, we predicted the sequence of the 3' extension using UNAFold.

The results indicated that the Δ*G* value for epegRNA4 with +AGA insertion is significantly lower than that with +AG insertion, decreasing from −9.6 to −12.5 kcal·mol^−1^ (Fig. 3B), suggesting a more stable RNA secondary structure. A similar trend was observed for epegRNA5 and epegRNA6, with Δ*G* differences of 0.3 and 1.2 kcal·mol^−1^, respectively (Fig. S2A,B). Across all three groups, the addition of a base pair generally resulted in lower Δ*G* values, indicating a more negative free energy state and a more stable RNA secondary structure.

Comparing the predicted RNA secondary structures of 3' extension sequences with different insertions, we observed that epegRNAs with +AGA insertion were more stable. The number of stems formed near the insertion sequence in epegRNA4 increased from 3 to 6 when the inserted sequence changed from +AG to +AGA (Fig. 3B, Orange). Similarly, epegRNA5 (+AGA ins) and epegRNA6 (+AGA ins) also exhibited an increase of two additional stems (Fig. S2A,B, Orange). This trend aligns with the predicted free energy values, where epegRNAs with higher free energy tend to form more stems, indicating a more stable secondary structure. However, this observation contrasts with our previously measured editing efficiency, where epegRNAs with more stable structures exhibited lower editing efficiency. This suggests that while increased structural stability may enhance pegRNA integrity, ultimately editing efficiency is reduced.

### Reducing structural complexity in epegRNAs enhances editing efficiency

To further validate that the observed decrease in editing efficiency was due to the structural complexity of the epegRNA with the +AGA insertion. Specifically, the formation of additional stem structures, we designed an alternative epegRNA with the same RTT and PBS length but replaced the original +AGA insertion with +CGC, which also encodes arginine (Fig. S1C,D).

According to computational predictions, this alternative epegRNA (epegRNA+CGC ins) exhibited higher free energy and a comparable number of stems to the epegRNAs with the +AG insertion (Fig. 3C). To assess its impact on editing efficiency, we performed ddPCR analysis. The results demonstrated that this substitution led to a modest improvement in editing efficiency, increasing from 4.68% to 7.95% (1.70‐fold) (Fig. 3D). This 1.7‐fold increase strongly supports the hypothesis that the prior decrease in editing efficiency was primarily attributable to the formation of more complex secondary structures in the epegRNA with the +AGA insertion.

## Discussion

Prime editing represents a major advancement in precision genome editing [26]. Although improved prime editor variants and optimized pegRNA designs have enhanced editing efficiency, challenges remain in both practical and clinical applications. In this study, we compared several prime editor variants and identified key features of pegRNAs that contribute to higher editing efficiency. To achieve this, we developed a FluoPEER.*PLN* R14del reporter cell line, enabling direct quantification of editing efficiency through mCherry fluorescence.

Our findings highlight the critical role of pegRNA secondary structure in determining prime editing efficiency. We initially demonstrated that epegRNAs with 16‐nt RTTs outperform their 14‐nt counterparts, suggesting that RTT length optimization is crucial for enhanced editing. Additionally, we systematically compared PE2, PE2max, PE2RnaseH^del^, and PE4, finding that while PE4 offers modest benefits by suppressing MMR, overall editing efficiency remains similar across variants.

A key challenge encountered was the reduced efficiency of +AGA insertions compared to +AG insertions. Our RNA structural predictions revealed that the additional nucleotide induces extended stem‐loop formations, increasing RNA stability but reducing prime editing efficiency. Prior studies support this observation, showing that longer stem structures in the 3' extension can lead to truncated reverse transcription products, ultimately compromising editing efficiency [7]. To validate this, we replaced the original AGA codon with CGC, which also encodes arginine but is predicted to form fewer stem structures. Our results showed that epegRNA+CGC ins partially restored editing efficiency compared to epegRNA+AGA ins, suggesting that pegRNAs with simpler secondary structures achieve higher editing efficiency. However, the editing efficiency of the +CGC variant did not fully recover to the level observed with +AG, despite exhibiting comparable free energy and predicted stem numbers. This result appears to challenge the notion that increased pegRNA stability alone accounts for reduced editing efficiency. A key distinction, however, is that the +CGC design required a 17‐nt RTT, whereas the +AG employed a 16‐nt RTT. Although a single‐nucleotide extension may seem minor, even slight increases in RTT length can alter the secondary or tertiary structure of the 3' extension or affect the processivity of reverse transcription [5]. These subtle structural or kinetic effects may contribute to differences in editing outcomes. Therefore, this observation highlights that pegRNA design should not only optimize secondary structure and thermodynamic stability but also carefully consider RTT length as an independent parameter influencing editing efficiency.

While our study demonstrates a structural and functional relationship of pegRNA for *PLN* R14del correction, several limitations should be considered. First, our experiments were conducted exclusively in HEK293FT cells, which may not fully recapitulate the editing efficiency in primary or patient‐derived cells. To address the translational potential of these findings, it is important to evaluate pegRNA performance in disease‐relevant cell types such as cardiomyocytes. Cardiomyocytes derived from human induced pluripotent stem cells (hiPSC‐CMs) offer a promising platform to assess editing efficiency in a more physiologically relevant setting [27]. These cells may differ substantially from HEK293FT cells in terms of chromatin architecture and the availability of repair machinery, potentially affecting both editing outcomes and pegRNA structural constraints [28]. Therefore, translating our pegRNA design principles to cardiomyocytes will be a critical next step in validating their broader applicability for therapeutic use. Second, the structural optimizations were evaluated for a single target sequence, and additional work is needed to assess the generalizability of our approach across different genetic conditions. Finally, while our study focuses on coding sequence corrections, future studies should investigate whether similar strategies are effective for editing regulatory or noncoding genomic regions.

Future studies should explore additional modifications to pegRNA structures, such as introducing chemically stabilized RNA motifs or engineering structural elements to enhance pegRNA stability and half‐life. Furthermore, expanding these optimizations to a broader range of genetic conditions will be essential to confirm their applicability to other gene‐editing contexts. Finally, validating these optimized pegRNA designs in patient‐derived cardiomyocytes will be a crucial step toward translational applications in genetic cardiomyopathies. Since the overall efficiency of prime editing remains suboptimal, further refinements will be necessary to achieve higher editing rates suitable for therapeutic applications.

## Conflict of interest

The authors declare no conflict of interest.

## Author contributions

BY, ZL, and JPGS conceptualized and developed the project. CJBSB performed cell sorting. MAD contributed to DNA extraction. QY contributed to plasmid construction. PAD, RS, ZL, and JPGS supervised the study. All authors participated in the writing‐review and editing of the manuscript and approved the final version.

## Supporting information


**Fig. S1.** Design and validation of epegRNAs and comparative analysis of ddPCR and FACS. (A) Agarose gel electrophoresis validation of epegRNA production. (A) consistent band of approximately 630 bp is observed for each of the 6 epegRNAs (epegRNA1‐6), confirming successful synthesis and amplification of the designed constructs. The molecular weight ladder on the right side of the gel serves as a size reference. (B) Schematic representation of the ddPCR and FACS methods for assessing editing efficiency. (C) Editing efficiency measured by ddPCR and FACS for three different epegRNAs with AG insertion (epegRNA4, epegRNA5, and epegRNA6). No significant difference (ns) was observed between the two methods across all tested conditions. Data are represented as mean ± SD (*n* = 3). (D) Schematic of epegRNA4 designed for the *PLN* R14del target, showing the specific sequences in the PBS (yellow) and RT template (brown) regions. The inserted bases are highlighted in red. (E) Agarose gel electrophoresis validation of epegRNA4 production. A consistent band of approximately 630 bp is observed for each of the 3 epegRNAs (+AG, +AGA, +CGC), confirming successful synthesis and amplification of the designed constructs. The molecular weight ladder on the right side of the gel serves as a size reference.

**Fig. S2.** The UNAFold‐predicted structures of the 3′ extension of epegRNAs with + AG and + AGA Insertions. (A and B)Predicted secondary structures of epegRNAs (epegRNA5, and epegRNA6) with +AG (left) and + AGA (right) insertions. The inserted nucleotides are indicated in red, and the orange regions represent stem structures that are predicted to form near to the desired edits.

**Table S1.** Primer sequences for PCR amplification of PLN R14del and epegRNAs.
**Table S2.** Spacer, scaffold, and extension sequences for all epegRNAs used in this study.
**Table S3.** Complete sequence of the FluoPEER.PLN R14del reporter plasmid.

## Data Availability

The data that support the findings of this study are available from the corresponding author or first author upon reasonable request.
