# Supplementary figures and images for: Insights into pegRNA design from editing of the cardiomyopathy‐associated phospholamban R14del mutation
Source: FEBS Lett. 2025 Jun 24;599(17):2543–54. doi: 10.1002/1873-3468.70097 (PMC12421704; doi:10.1002/1873-3468.70097)

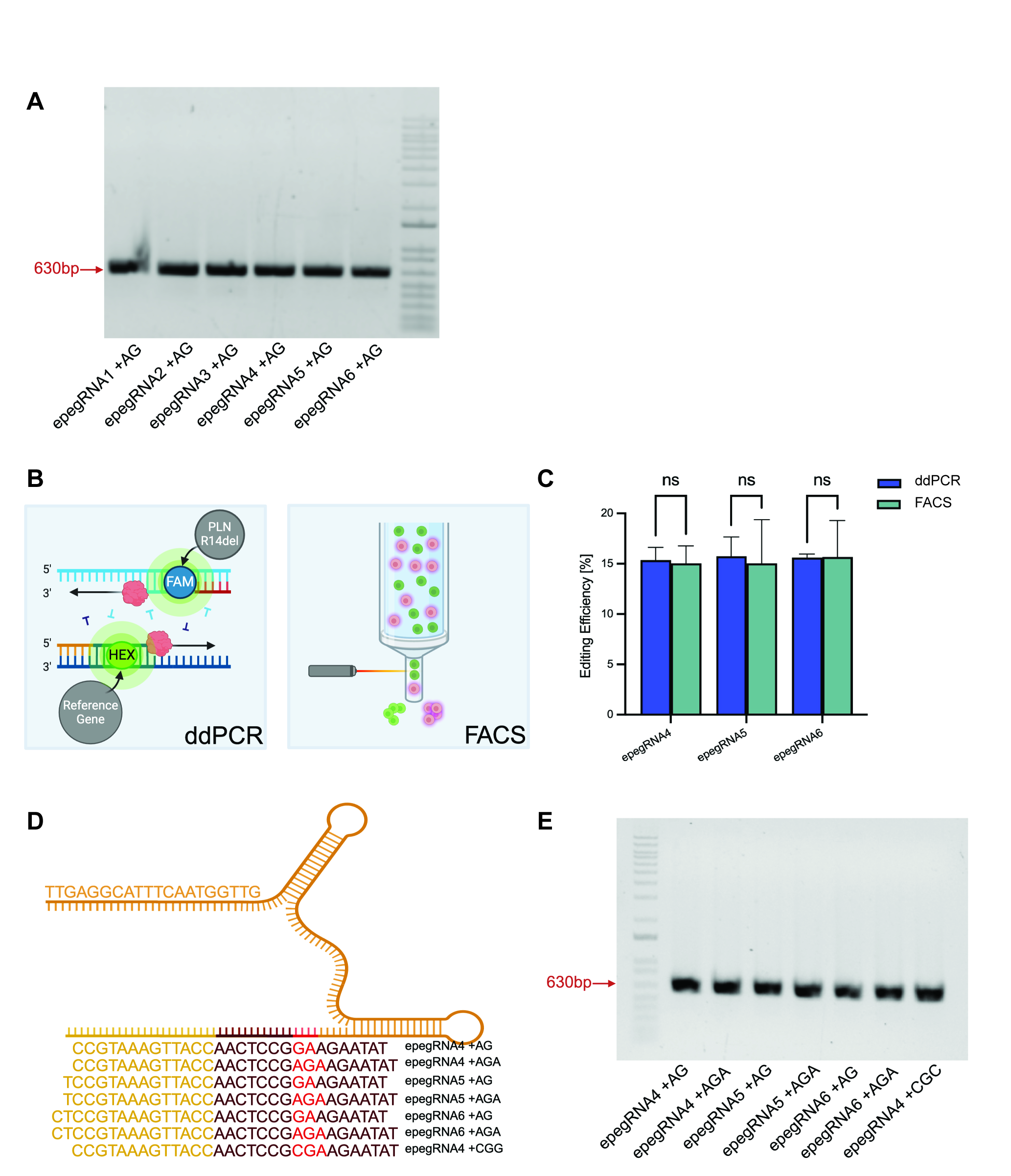

Supplement: Supplementary file 1 — Fig. S1. Design and validation of epegRNAs and comparative analysis of ddPCR and FACS. (A) Agarose gel electrophoresis validation of epegRNA production. (A) consistent band of approximately 630 bp is observed for each of the 6 epegRNAs (epegRNA1‐6), confirming successful synthesis and amplification of the designed constructs. The molecular weight ladder on the right side of the gel serves as a size reference. (B) Schematic representation of the ddPCR and FACS methods for assessing editing efficiency. (C) Editing efficiency measured by ddPCR and FACS for three different epegRNAs with AG insertion (epegRNA4, epegRNA5, and epegRNA6). No significant difference (ns) was observed between the two methods across all tested conditions. Data are represented as mean ± SD (n = 3). (D) Schematic of epegRNA4 designed for the PLN R14del target, showing the specific sequences in the PBS (yellow) and RT template (brown) regions. The inserted bases are highlighted in red. (E) Agarose gel electrophoresis validation of epegRNA4 production. A consistent band of approximately 630 bp is observed for each of the 3 epegRNAs (+AG, +AGA, +CGC), confirming successful synthesis and amplification of the designed constructs. The molecular weight ladder on the right side of the gel serves as a size reference. [file FEB2-599-2543-s003.tif]

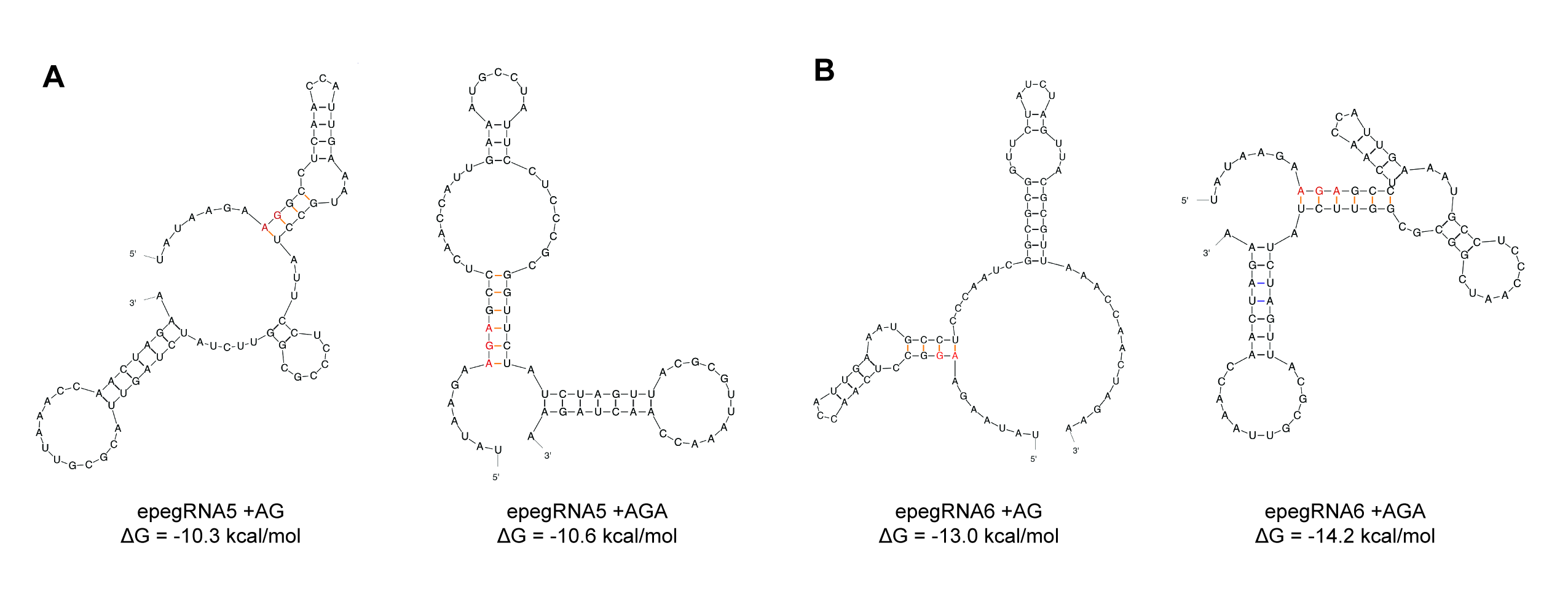

Supplement: Supplementary file 2 — Fig. S2. The UNAFold‐predicted structures of the 3′ extension of epegRNAs with + AG and + AGA Insertions. (A and B)Predicted secondary structures of epegRNAs (epegRNA5, and epegRNA6) with +AG (left) and + AGA (right) insertions. The inserted nucleotides are indicated in red, and the orange regions represent stem structures that are predicted to form near to the desired edits. [file FEB2-599-2543-s002.tif]
